# A flexible and low power telemetric sensing and monitoring system for chronic wound diagnostics

**DOI:** 10.1186/s12938-015-0011-y

**Published:** 2015-03-01

**Authors:** Nasir Mehmood, Alex Hariz, Sue Templeton, Nicolas H Voelcker

**Affiliations:** School of Engineering, University of South Australia, Adelaide, SA, 5001, , Australia; Royal District Nursing Service, Adelaide, SA 5035 Australia; Mawson Institute, University of South Australia, Adelaide, SA 5001 Australia

**Keywords:** Chronic wound monitoring, Telemetric sensing, Chronic wound management, Wound diagnostic system

## Abstract

**Background:**

Non-healing chronic wounds, such as venous leg ulcers, can be monitored non-invasively by using modern sensing devices and wireless technologies. The development of such a wireless diagnostic tool may improve chronic wound management by providing evidence on efficacy of treatments being provided. This paper presents a low-power portable telemetric system for wound condition sensing and monitoring. The system aims at measuring and transmitting real-time information of wound-site temperature, sub-bandage pressure and moisture level from within the wound dressing.

**Methods:**

Commercially available non-invasive temperature, moisture, and pressure sensors are interfaced with a telemetry device on a flexible 0.15 mm thick printed circuit material to construct a light-weight, non-invasive, biocompatible, and low-power sensing device. The real-time data obtained is transmitted wirelessly to a portable receiver which displays the measured values. The performance of the whole telemetric sensing system is validated on a mannequin leg using commercial compression bandages and dressings. A number of trials on a healthy human volunteer are performed where treatment conditions were emulated using various compression bandage configurations.

**Results:**

A reliable and repeatable performance of the system is achieved under compression bandage and with minimal discomfort to the volunteer. The system is capable of reporting instantaneous changes in bandage pressure, moisture level and local temperature at wound site with average measurement resolutions of 0.5 mmHg, 3.0% RH, and 0.2°C respectively. Effective range of data transmission is 4–5 m in an open environment.

**Conclusions:**

A flexible and non-invasive sensing system is developed to acquire and wirelessly transmit wound parameters from within a compression bandage and wound dressing worn on a human limb. Pre-clinical results on a healthy human subject suggest its clinical usability and value to health practitioners. However, further performance evaluations of the device on a wider population of healthy human subjects and on patients with chronic wounds are required to confirm its medial usefulness and to quantify its real impact on chronic wound management.

## Introduction

Chronic wounds, such as venous leg ulcers and diabetic foot ulcers, are posing a financial threat to the healthcare systems in the world [1,2]. A current estimate shows the economic cost of woundcare activities in the world is distributed as 15-20% materials, 30–35% nursing time and more than 50% as hospitalization time [3]. The woundcare cost in Australia only is estimated at $ 2.5 billion per year with more than 4000 limb amputations [4]. A 2001 study indicated that chronic wounds were a major cause of morbidity, affecting more than 1% of UK population and with treatment cost of at least £1 billion [5]. During 2006–07, chronic wounds were affecting 3–6 million people in the USA with a total cost of treatment estimated at more than $3 billion annually [6,7]. In 2012, approximately 7 million people suffered from chronic wounds in the USA, and the cost for their treatment was estimated at almost $25 billion annually [8].

The most effective and economical treatment of wounds is covering them with a suitable dressing or bandage in order to protect damaged skin from external effects such as microorganism attacks [9]. For certain chronic wounds such as venous leg ulcers, appropriate compression bandages are applied to increase the healing rate [10,11]. These bandages may be retention (low pressure), light support (medium pressure) or compression (high pressure) bandages. The method of applying compression bandages on the affected limb is very important as the efficacy and maintenance of sub-bandage pressure depends on it [12]. Compression bandages can produce a pressure up to 60 mmHg at the ankle (extra high pressure), while the recommended high pressure value is 40 mmHg at the ankle [10,12]. Depending on the applied pressure range and the type of bandage used, the sub-bandage pressure may vary significantly during the physical movement of the patient, thus affecting the healing rate [13]. In addition to compression bandages, healing rates may also be increased by managing moisture produced by the wound (exudate) through moisture-retentive dressings such as Anasept^®^ (hydrogel) and Hydrocolloids [14,15] for wounds with low exudate and dressings such as Allevyn^®^ (foam) or Melgisorb^®^ (calcium alginate) for wounds with moderate to high exudate. In addition to moisture levels, the temperature and pH under the dressing may change as a result of an infection [16,17]. Unfortunately, these parameters associated with the dressings are not currently monitored in clinical practice. There is an opportunity for advanced sensor technologies to contribute to improved wound monitoring and diagnostics.

A highly accurate (0.2°C accuracy) RFID-based skin temperature monitoring system was devised by Matzeu et al. [18]. However, the system lacked proper clinical trials using wound dressings. Moser and Martin [19] presented a flexible platinum-based miniaturized temperature sensor to operate within 0–400°C range. For this device too, no testing on humans was performed. McColl et al. [20] developed an impedance sensor moisture monitoring system to be used with wound dressings. Following this, Ohmedics^©^ developed a clinically-proven moisture monitoring device called WoundSense^®^ [21]. However, this device is not designed to stay within the dressing for continuous moisture sensing. Khaburi et al. [22] reported a force sensors-based pressure-mapping bandage prototype to measure pressure at various points on a leg mannequin. A wearable sub-bandage pressure measurement system is reported by V. Casey et al. [23] for real-time pressure measurements with a wired connection between the flexible sensor and the display module. To the best of our knowledge, no device exists for continuous and non-invasive monitoring of sub-bandage pressure for wounds. However, miniaturised pressure sensors have been fabricated and used in other applications including intracranial pressure [24,25], intraocular pressure [26], spinal plates pressure [27] and for general in vivo applications [28-30]. In other reported literature, researchers have proposed integrated wound monitoring systems using wireless data transmission [31,32]. However, those devices have not been tested or utilised in a wound environment and their wireless transmission range was confined to a few millimetres, thus restricting the practical use of the devices.

In this paper, we demonstrate a flexible wireless telemetric system for continuous sensing and monitoring of the wound environment, proposed in our earlier review article [33]. Preliminary results indicate that the system is capable of measuring and transmitting real-time information on temperature, moisture, and sub-bandage pressure from under the bandage or within the wound dressing at programmable transmission intervals [34]. The selection of sensors and their calibration processes have been discussed in our recent article [35]. The sensing system is fabricated on a flexible printed circuit material, while the sensors are micro-sized and flexible, thus making the system minimally invasive to wounds and the human body. The receiver is portable with the capability to receive data accurately within a distance of 4–5 meters. The system has been tested on a human volunteer using various compression bandages and moisture-retentive dressings. The results from these trials confirm the clinical utility of this system in a wound environment.

## Methods and materials

For chronic wound monitoring application, the diagnostic device is required to perform reliably in a delicate environment involving human skin and wound fluid. In addition to satisfy the essential criteria of flexibility, protection from wound chemicals, and bio-compatibility, the device needs to fulfil certain performance requirements as well, which sets the foundation for minimum measurement resolutions. For meaningful temperature measurements, the device must be able to detect changes in temperature of less than ± 0.5°C. The case with pressure and moisture is different. Although, the aim of compression bandages and stockings is to maintain a constant sub-bandage pressure at certain positions on limb, however, it may be anticipated that a ± 5 mmHg variation in bandage pressure would not have a significant impact on wound healing. Similarly, a ± 5% RH resolution could be expected for moisture measurements. The wireless device for this application does not need to transmit continuously as the wound conditions do not change abruptly. A complete packet of information transmitted twice an hour would be sufficient. All the user and medical requirements for the wound sensing system are listed in Table 1. The proposed sensing system satisfies all of the above mentioned medical and performance requirements as explained in the following sections.

**Table 1:** **Requirement specifications for the developed chronic wound monitoring system**

**Parameters**	**Type/** **category**	**Specifications**
Sensors	Temperature	At least 01 sensor, 0.5°C resolution, miniature and flexible
	Moisture	At least 01 sensor, 5% RH resolution, miniature and flexible
	Pressure	At least 01 sensor, 5 mmHg resolution, miniature and flexible
System size	NA	Small enough for placement within a compression bandage
Power consumption	NA	Must be kept as low as possible for long-term operation
Transmission	Protocol	Any suitable protocol e.g. Zigbee^®^, Bluetooth^®^, WiFi^®^
	Range	Suitable to receive data in a clinical setup
	Power	Within the safe exposure limits for human body
	Frequency	2.4 GHz band to keep the components’ size smaller
	Interval	Should be kept high to save battery power, e.g. 10 minutes
Ergonomics	NA	Must be flexible, bio-compatible, and non-invasive
Response time	NA	Less than or equal to a second

### Selection and calibration of sensors

For wound monitoring application, the sensors and their assemblies need to be biocompatible and minimally invasive to the human body, as the sensors would be placed within a wound dressing or compression bandage over a human limb [36]. Having metallic inflexible structures of sensors and circuit components would create discomfort to the patients. It has been revealed through online surveys that the majority of available sensors do not qualify for this particular application because of their large size, invasive structure, complex principle of measurement, and the need for additional on-board circuit components for operation. Table 2 below lists a few such sensors along with the reasons of their unsuitability for wound diagnostics.

**Table 2:** **A partial list of commercially available temperature**, **pressure and moisture sensors**

**Sensor type**	**Model no.**	**Dimensions** **(mm)**	**Reasons of unsuitability**
Pressure	Honeywell^®^ 170PC	21.5 × 21.5 × 34.3*	Invasive to human body due to large size. Fluid flow is required to create pressure.
Pressure	Honeywell^®^ 19C015A7	19.0 × 35.6^#^	Invasive to human body due to metallic port and large size.
Pressure	Measurement Specialities™ FC22	34.0 × 16.8^#^	Invasive to human body due to large metallic structure.
Pressure	Measurement Specialities™ MS5540C	6.4 × 6.4 × 3.0*	Invasive to human body due to hard metallic structure.
Pressure	Interlink Electronics FSR406	38 × 38 × 0.5	Flexible, non-invasive, and bendable structure. Works on the principle of impedance change with applied pressure.
Moisture	Honeywell^®^ HIH403X/503X Series	8.65 × 4.20 × 3.0*	Less invasive to human body due to small size. Air samples are required for moisture measurement.
Moisture	Silicon Laboratories Inc. Si7005	4.0 × 4.0 × 1.5*	Less invasive to human body due to small size. Air samples are required for moisture measurement. Needs additional on-board circuits to operate.
Moisture	TDK Corporation CHS-SS Series	25 × 10 × 5.0*	Invasive to human body due to relatively large size. Air samples are required for moisture measurement.
Moisture	Multicomp HCZ-D5	10 × 5.0 × 0.5*	Miniature size, comparatively less invasive. Works on the principle of impedance change with the moisture level.
Temperature	Texas Instruments LM62	3.0 × 1.4 × 1.1*	Suitable for placement within the dressing. Accuracy ±3°C.
Temperature	Texas Instruments LM35DM	6.5 × 5.4 × 2.0*	Dimensions are not suitable for placement within the dressing. Accuracy ±0.5°C.
Temperature	Texas Instruments LM94021	2.15 × 2.4 × 1.1*	Dimensions suitable for placement within the wound dressing. Accuracy ±1.5°C. Gain can be controlled digitally.

*Length x width x height in mm, # diameter x length in mm.

These sensors are listed based on their available minimum size.

Sensors need to be carefully selected, calibrated and characterized for the intended environment in order to capture reliable information. Any error in the sensor’s measurement would spread through the whole system and in some scenarios might get amplified. This would create ambiguities and false diagnosis by clinicians and health practitioners. For wound-site temperature monitoring, we chose LM94021B (Texas Instruments, USA) temperature sensor for its small size and reliable performance. This ultra-low power sensor typically consumes just 9 μA current at a rated 5.0 V supply voltage. With a size of 2.15 mm × 2.40 mm × 1.1 mm (L x W x H) and a nominal accuracy of ±1.5°C in the temperature range 20–40°C [37], the sensor is quite suitable for our wound monitoring system.

For moisture sensing, Honeywell HIH4030 piezo-electric and Multicomp’s HCZ-D5 piezo-resistive moisture sensors were calibrated, characterized, and used with a prototype wireless sensing system. The piezo-resistive sensor (HCZ-D5) was found the most suitable sensor for this application because of its small size (10 mm × 5 mm × 0.5 mm). The sensor was calibrated and characterized using a dedicated experimental setup. An interface circuit was also designed to properly operate the moisture sensor.

For sub-bandage pressure measurement, we used the Interlink Electronics’ FSR406 piezo-resistive pressure sensor with a square sensing area of 38 mm × 38 mm. This sensor is non-invasive, flexible and is only 0.5 mm in thickness. The pressure sensor was calibrated using a clinical-grade pressure meter HPM-KH-01 for validation of pressure measurements up to 40 mmHg which is regarded as the desired value for high sub-bandage pressure [12]. An interface circuit was also designed to properly operate the pressure sensor. A commercial compression bandage system (Coban™ 2) was used to create pressure over the sensor placed on a mannequin leg.

### Flexible wireless sensing system

A number of System-on-Chip (SoC) devices are commercially available with telemetry functions. Most of these devices use standard wireless transmission protocols, such as WiFi^®^, Bluetooth^®^, Bluetooth Low Energy^®^, and ZigBee^®^. Some examples of such SoCs are CC2530/31 (ZigBee), CC2540/41 (Bluetooth Low Energy), CC2560/64 (Bluetooth), CC3000/3100/3200 (WiFi), EM358x (ZigBee), 88MZ100 (ZigBee), ATMega128RFA1 (ZigBee) etc. In our proposed system, we have chosen ZigBee^®^ for its simplicity, reasonable range, and low power operations. For this purpose, we have used Atmel’s ATMega128RFA1 RF transceiver in our sensing system [38]. The transceiver is of small size (9 mm × 9 mm × 1 mm) and operates at 2.45GHz ISM (industrial, scientific, medical) frequency band using IEEE 802.15.4 ZigBee^®^ protocol. It has a−100 dBm sensitivity and a 3.5 dBm programmable output power. It also contains a programmable serial interface and a 10-bit analog-to-digital (ADC) converter, thus the hardware overhead would be minimized.

**Figure 1:**
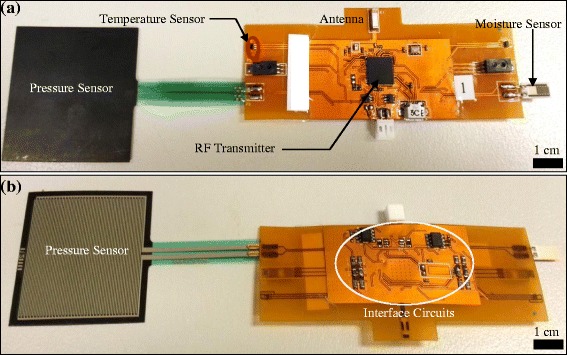
**The developed flexible sensing system. (a)** Photo of the wireless sensing system fabricated on a 0.15 mm thickness 2-layer flexible printed circuit board. The diagram shows various parts of the sensing system **(b)** Rear side of the sensing system showing the voltage divider network used as interface circuits between the sensors and the ADC.

For placement within the dressing, the wireless sensing system was designed and fabricated on a 0.15 mm 2-layer flexible printed circuit board (Figure 1(a)). In order to protect the delicate components within the bandage measurement environment, and to avoid any contact of circuit materials with the skin, a transparent biocompatible polydimethylsiloxane (PDMS) was coated over the exposed circuit components. The sensors were connected to port F (ADC input port) of the transceiver through the customised interface circuit. A balance-unbalance (Balun) device [39] (Johanson Technology^©^ P/N. 2450FB15L0001 recommended for ATMega128RFA1) and a 2.4GHz chip antenna [40] (Johanson Technology^©^ P/N. 2450AT43B100) were used for accurate radio operations. Special care was taken while routing the RF circuit tracks and the RF components on the circuit board following the manufacturers’ (Atmel^©^ and Johanson Technology^©^) guidelines. Exactly the same components and routing strategy were adopted on the receiver side. A firmware program was designed in C++ and is implemented in the transceiver devices on both circuits i.e. transmitter (TX) and receiver (RX), using the Atmel’s AVR Studio 6.0 software tool.

The information captured by the sensors was first converted into digital format and then stored into the TX frame buffer register. The first byte of TX frame buffer register was written with frame length information followed by the sensors’ data [38]. Before transmitting, the transmitter was passed through a set of pre-defined states i.e. RESET→TRX_OFF→PLL_ON→TX_ON. After transmitting one packet of information, the transmitter was turned off (TRX_OFF) to save battery energy.

The net weight of the sensing system is 1.938 g without sensors, 6.709 g with all sensors attached and, 9.740 g with sensors and an alkaline battery having dimensions 14 mm x 10 mm (length x diameter). The nominal and maximum current consumptions of the sensing system were measured as 13.58 mA and 17.4 mA, respectively. Hence, the peak power consumption of the sensing system is 57.4 mW at 3.3 V supply voltage. As most of the power is consumed in the RF circuits, reducing the RF switching frequency could save a considerable battery power. In a chronic wound monitoring application, even a very low data transmission rate (e.g. one packet of information per quarter an hour) would be sufficient because changes in wound parameters are very slow. A high data transmission rate would only mean a high switching rate of RF components resulting in unnecessary loss of battery power.

### Interfacing the sensors with the radio transmitter

The result of ADC conversion was stored in two 8-bit registers; ADCH for high byte and ADCL for low byte. The maximum reference voltage for the ADC was 1.8 V, which was internally generated and stabilized. All the sensors in our system work at a 5.0 V supply for all input values exceeding its reference voltage and would not be able to detect any input signal above 1.8 V. This problem was resolved by reducing the ADC input voltage range such that the maximum input voltage of any sensor was less than the ADC reference voltage. For this purpose, a simple voltage divider circuit was designed using two surface mount resistors R_1_ and R_2_ for each sensor (Figure 1(b)).

The voltage divider circuit necessitates a proper impedance matching with the sensors. The temperature sensor LM94021 consumes almost 9 μA current at 5.0 V. So, its impedance was nearly 555 kΩ. In order to safely reduce the output voltage, we have chosen 750 kΩ standard values for both resistances in Figure 1(b) providing a voltage division ratio of two. The active moisture sensor HIH4030 operates at 5.0 V, consuming almost 200 μA of current, thus producing an input impedance of 25 kΩ. For this sensor, we have used R_1_ = 46 kΩ and R_2_ = 25 kΩ, resulting in a voltage division ratio of 2.84. Using the same principle for the pressure sensor FSR406, the resistance values were chosen as R_1_ = 56.2 kΩ and R_2_ = 46.4 kΩ, resulting in a voltage division ratio of 2.21. For the passive moisture sensor HCZ-D5, the values of R1 and R2 were 46 kΩ and 25 kΩ, respectively. The values of R_1_ and R_2_ might be adjusted to any other values provided they generate the same division ratio and their combined effect does not overrule the impedance matching criteria. It may be noted that the moisture level was measured with the passive sensor only.

### Information display module

The transmitted data in ZigBee^®^ 802.15.4 protocol was received by the handheld receiver, processed by the T6963C LCD controller, and displayed on a 10 cm x 8 cm LCD screen [41]. The LCD controller was programmed through the receiver module (ATMega128RFA1) by sending the appropriate commands with required timings as recommended in the datasheet of T6963C [42]. The first transmitted byte consists of frame length information followed by the temperature, moisture and sub-bandage pressure data, respectively. Upon reception, the sensor’s data was stored in a 128-byte frame buffer register. This data was then sent to the LCD controller (T6963C) through a parallel interface for processing and display. The LCD controller was also programmed to display the device ID (TX device), frequency channel (2405–2480 MHz in 5 MHz steps), and received signal strength (i.e. RSSI) in dBm.

The TX device ID may be used as a unique identifier for the patient under observation. In a hospital environment where a number of patients would be using these devices, unique frequency channels may be allocated to all devices to avoid any interference between devices or corruption of data. The RSSI level could be indicative of how trustworthy the information of measured parameters is. Information associated with much lower RSSI levels (e.g. -90 dBm) may be discarded on the grounds that the TX device might be too far away from the RX or there might be some obstacle between them, thus inhibiting a reliable communication link. The experiments that involve a population sample for the purpose of evaluating the device from a clinical perspective are underway, they have received ethical approval, and will be the subject of a forthcoming paper. The experiments included in the present paper are carried out by a volunteer for the purpose of scientific evaluation of the device performance. They are not intrusive, and do not interfere with the bodily functions of the subject. They are no different than wearing a heart monitor or a blood-pressure monitor and recording the response as a function of activity. To that effect there is no ethical approval needed, and none were sought.

## Experiments and results

The developed wireless wound sensing system was first tested in a room environment using commercial compression bandages and a mannequin leg. The compression bandages used for the experiments consisted of AMS Bi-Flex^®^ elastic bandage, Hartmann Lastodur Light^®^ long-stretch bandage, 3 M Coban™ 2 two-layer, and Hartmann Lastolan^®^ short-stretch bandages. Adjunct components used in the application of compression bandage systems such as Soffban^®^ undercast padding and Idealcrepe^®^ crepe retention bandage were also used. Using the experimental setup, a number of experiments were performed using the mannequin leg in a room environment. The sensing system was placed flat on the central portion of the mannequin to avoid any damage to the components from bending. After the application of dressing, distilled water was sprayed over the bandage in the proximity of the moisture sensor. The measurements were taken with an interval of 5 minutes by the handheld receiver placed 3 m apart in line of sight to the wireless transmitter. The experiment was performed at 25°C. The observations are reported in Table 3 using that the average values of received energy level, temperature, and sub-bandage pressure were calculated as −79.92 dBm, 23.21°C and 15.14 mmHg, respectively. The average errors in temperature, moisture, and sub-bandage pressure measurements were calculated as 0.93°C, 3.0% RH, and 1.50 mmHg, respectively. However, the error in sub-bandage pressure measurements is expected to increase on human leg because of flexible morphology and muscle movements. The errors in moisture and temperature measurements are immune to these factors.

**Table 3:** **Results of initial experiments with the wireless sensing system in an open environment**

**Time** **(minutes)**	**Energy** (dBm)*	**Temperature** **(°C)**	**Moisture** **(%RH)**	**Pressure** **(mmHg)**
T0	−81	25	5	19
T0 + 5	−81	24	3	18
T0 + 10	−79	23	7	17
T0 + 15	−79	23	9	17
T0 + 20	−81	23	11	17
T0 + 25	−78	23	12	16
T0 + 30	−81	23	13	16
T0 + 35	−79	23	14	16
T0 + 40	−78	23	14	16
T0 + 45	−81	23	16	16
T0 + 50	−82	23	17	15
T0 + 55	−79	23	17	15
T0 + 60	−80	23	16	15
T0 + 65	−80	23	16	15

*Receiver sensitivity is −100 dBm. Energy represents the energy detected during packet reception.

The manufactured wound sensing and monitoring system (Figure 1) was extensively tested for its performance on a healthy volunteer. The objectives of these trials were to validate the performance of the sensing system under various clinical scenarios and to determine the optimum placement of the system and sensors on the human body. These scenarios were realized using different compression-bandage systems and various possible postures, both believed to influence the real-time measurements. These trials and their results are discussed in detail in the following sections.

### Verifying reliability of measurements

**Figure 2:**
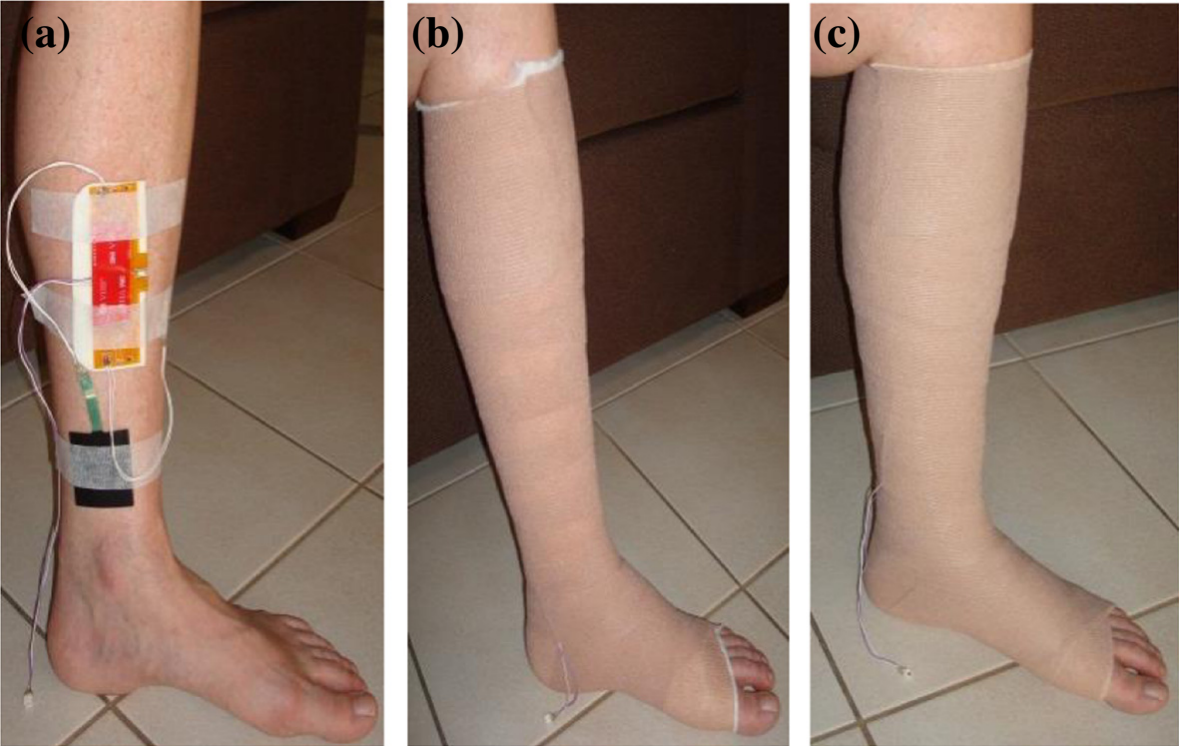
**Sensing system under a compression bandage.** **(a)** Sensing system and sensors in place over the leg prior to bandage application **(b)** 4-layer compression bandaging in place for pressure measurements **(c)** 2-layer inelastic bandaging in place for pressure measurements.

**Figure 3:**
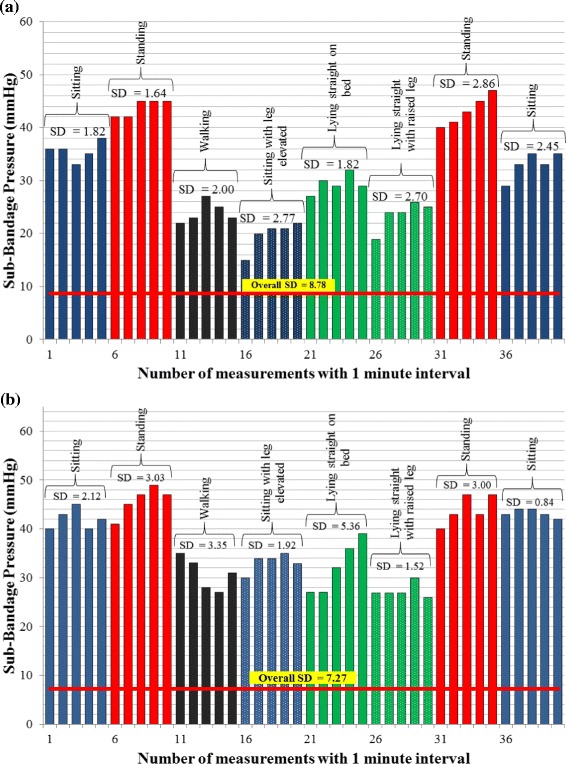
**Experimental results of sub-bandage pressure measurements.** Graphical plots of pressure measurements during the first trial using **(a)** 4-layer compression bandage system. **(b)** 2-layer compression bandage system. The measurements are recorded in various routine movements and postures.

In the first experiment the reliability of measurement results was tested, particularly the sub-bandage pressure measurements as they are prone to variations due to body movements. The sensing system was covered with a transparent adhesive silicone gel on both sides (Figure 2(a)). The trial was performed using a 4-layer compression system Profore^®^ (Smith & Nephew^©^) as well as a 2-layer inelastic bandage system Coban™ 2 (3 M^©^) for sub-bandage pressure measurements (Figure 2(b-c)). The bandages in all experiments were applied by a Wound Management Nurse Practitioner adept in managing chronic wounds and bandaging techniques. The sub-bandage pressure and moisture measurements were performed in two separate experiments. During the sub-bandage pressure measurements, the moisture sensor was placed directly on exposed skin with the sensor facing the skin while the pressure sensor was placed over the flat area of the exposed skin, directly above the medial malleolus (ankle), with the sensor facing the skin. Initial measurements were taken in each experiment prior to applying bandages in order to find and nullify any offset readings. The bandages were applied in accordance with the manufacturer’s instructions, achieving 40 mmHg starting pressure. The measurement results are plotted in Figure 3(a) (4-layer) and Figure 3(b) (2-layer), at one minute interval using various common postures. Variations in sub-bandage pressure readings are measured by calculating the standard deviation (SD), shown as a red horizontal line in graphs with sub-bandage pressure measurements.

**Figure 4:**
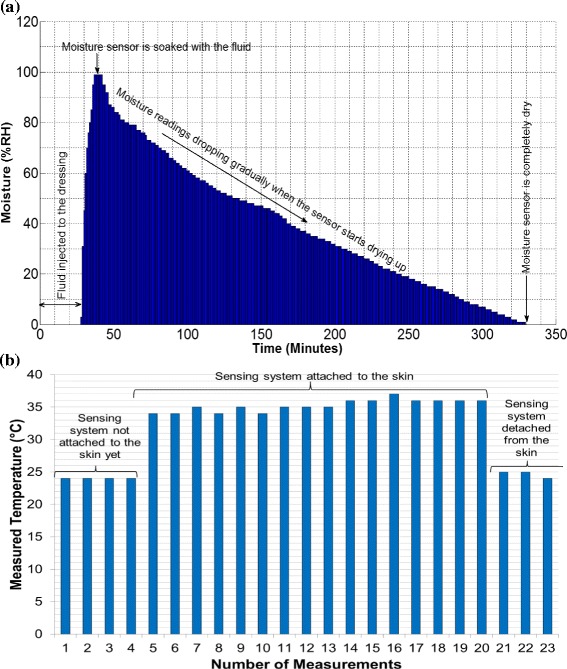
**Experimental results of moisture and temperature measurements. (a)** Graphical plot of moisture values measured using moisture-retentive dressing. Fluid was injected into the dressing through micro-volume extension tubing. The graph indicates a natural rise and fall of moisture level over time **(b)** Graph showing temperature measurements using the flexible sensing system. The graph shows almost constant readings of the room and the skin temperatures.

As the moisture and the temperature measurements are independent of posture changes, different setups were used from those used for sub-bandage pressure measurement. For moisture measurements, a moisture-retentive foam dressing Allevyn™ Adhesive (Smith & Nephew) was used. A small slit was made to insert micro-volume extension set tubing in one corner, and another slit was made to insert the moisture sensor in the opposite corner of the dressing. Since the sensor was dry, the measured moisture level was zero. Fluid was then injected through the tubing after every five minutes until the sensor was soaked and started providing moisture values. After that, the dressing was placed upside down so that the fluid moved away from the sensor until it was completely depleted of moisture. Moisture measurement results are plotted in Figure 4(a).

For temperature measurements, the sensing system was placed over the lower leg area with the temperature sensor facing skin, and then various temperature readings were recorded continuously for more than 15 minutes. The experiment was performed at 23°C room temperature, and with 40% humidity. The temperature measurements are plotted in Figure 4(b). The average value of the measured skin temperature was 35 ± 1°C while the standard deviation (SD) was 0.93°C for skin temperature measurements.

The sub-bandage pressure measurement results (Figure 3) for both bandages have shown a reliable and repeatable performance of the sensing system under required sub-bandage pressure. In each posture, five consecutive pressure readings were taken over a period of 5 minutes. For the 4-layer bandage system, the maximum deviation (SD value) observed was 2.86 mmHg during standing, while the same for the 2-layer bandage system was 3.35 mmHg during walking. The moisture level measurement results (Figure 4(a)) have also shown a reliable system performance. A steep rise in moisture level was observed as the injected fluid reached in proximity of the sensor. After that, a gradual and consistent drop in the measured moisture level was observed as the sensor was placed in an upward position to allow backward flow of the fluid. In about six hours, the moisture level dropped to zero when the sensor was completely depleted of the fluid. Thus, the first trial concludes that the measurement of temperature, moisture, and sub-bandage pressure with the developed sensing system is reliable, repeatable and consistent with the changes in experimental conditions.

### Measurements at ankle level with a 4-layer bandage

**Figure 5:**
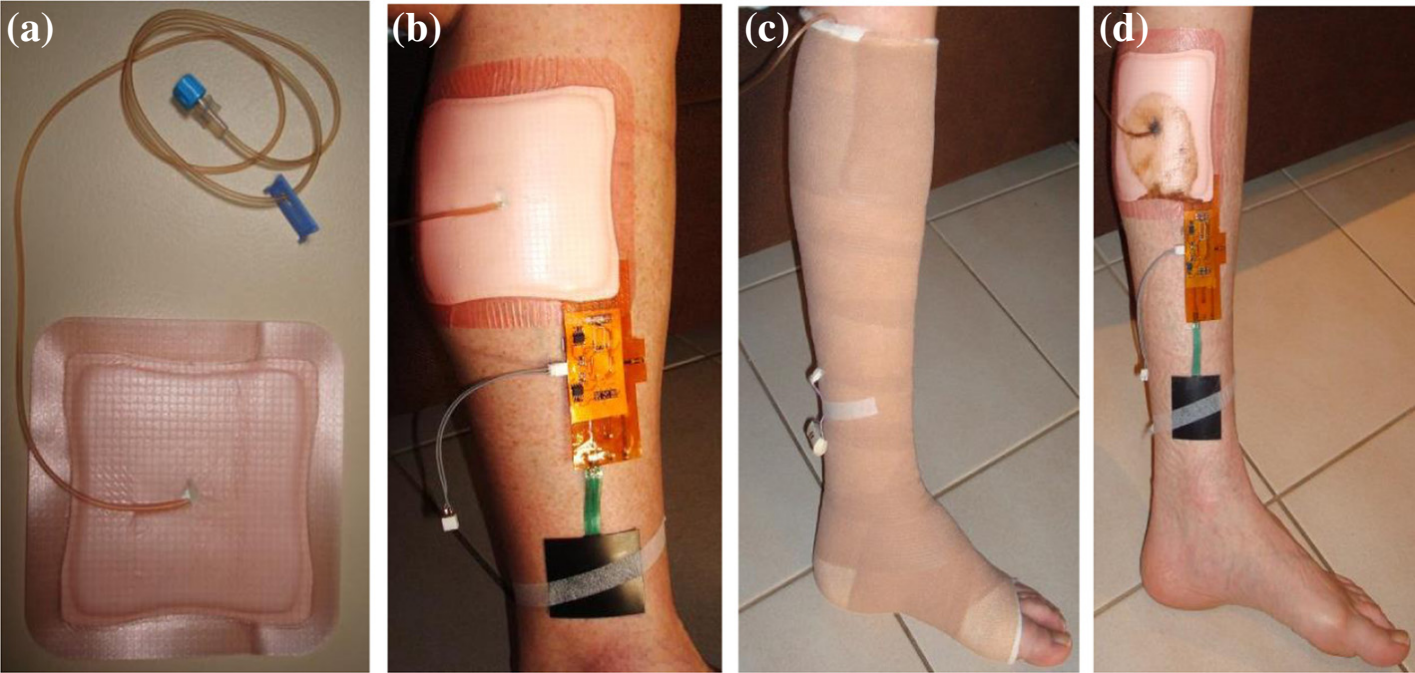
**Sensing system under a 4-layer compression bandage. (a)** Allevyn™ dressing with micro-volume extension set in place prior to application. **(b)** Dressing and sensors in place on leg prior to bandage application. **(c)** Bandaging in place immediately following application. **(d)** Dressing and sensors in place immediately following removal of bandages.

**Figure 6:**
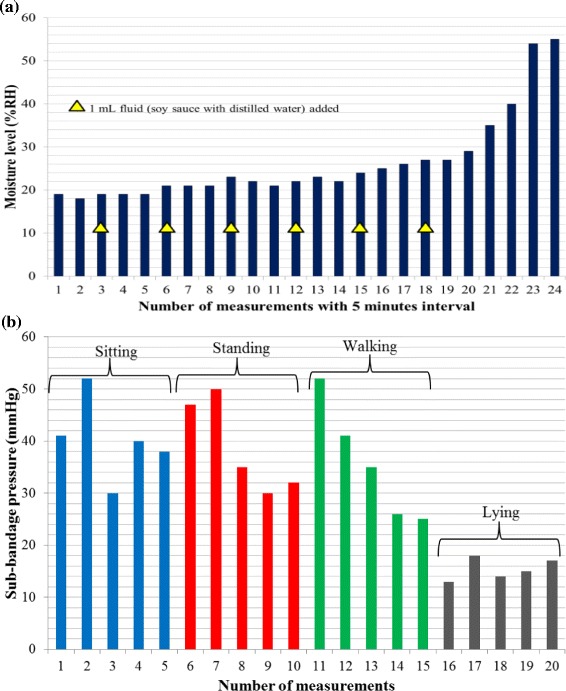
**Measurement results using a 4-layer compression bandage.** Graphical plots of the measured values of **(a)** moisture **(b)** sub-bandage pressure for experiments at ankle.

In this trial, a 4-layer bandage system known to apply 40 mmHg at ankle was used. Bandages were applied as per manufacturer’s instructions. A small slit was made in the outermost cohesive bandage (layer 4) to allow battery connection. Allevyn™ Adhesive (Smith & Nephew) 12.5 cm™ 12.5 cm dressing was applied to the lower calf and a small cut was made in the back of the dressing (Figure 5(a)). A micro-volume extension set tubing was attached to the dressing and the slit sealed with film tape to allow injection of fluid (As genuine wound fluid was not available soy sauce diluted with water was used). The fluid was injected under the dressing to mimic wound exudate and soy sauce was chosen so that the spread of fluid was visible during experiments. The sensing system was then attached to the lower leg (Figure 5(b)). The pressure sensor was placed proximal to the medial malleolus between exposed skin and bandages. The moisture sensor was placed at the bottom corner of the Allevyn™ dressing between the exposed skin and the dressing. The results of second trial are plotted in Figure 6. The average value of temperature during this trial was 33 ± 1°C. The moisture values were increasing gradually as more fluid was injected to the dressing until the sensor was soaked with the fluid, the point from where the readings started rising up (readings 21–24 in Figure 6(a)). The sub-bandage pressure values (Figure 6(b)) were dependent on posture, being higher during walking and standing and lower during sitting or lying. It can also be observed from the graph that the pressure readings were consistently dropping over time. This phenomenon can be attributed to loosening of bandage layers with movement.

## Discussion

In the first trial, the measurements for pressure, moisture, and temperature were taken separately using distinct experimental setups. The measurements were reliable and consistent with the applied conditions as can be observed in Figure 3, Figure 4(a), and Figure 4(b) for sub-bandage pressure, moisture, and temperature respectively. The sub-bandage pressure values are expected to fluctuate with the movement, and the graphs (Figure 3(a) and (b)) have verified this phenomenon. The overall fluctuation of sub-bandage pressure values measured as standard deviation was 8.69 mmHg for the 4-layer and 7.27 mmHg for the 2-layer bandage system. The postures were changed in a cyclic fashion (i.e. starting from ‘sitting’ and ending in ‘sitting’) to isolate the source of fluctuations in measurements.

The average sub-bandage pressure values recorded during the first and last ‘sitting’ postures for the 4-layer were 35.6 mmHg and 33.0 mmHg respectively for the 4-layer bandage, and were 42.0 mmHg and 43.2 mmHg respectively for the 2-layer bandage. Similarly, the average sub-bandage pressure values during both ‘standing’ postures were recorded as 43.8 mmHg and 43.2 mmHg respectively for the 4-layer bandage, and were 45.8 mmHg and 44.0 mmHg respectively for the 2-layer bandage. The twin average values during respective postures for each bandage system were pretty close to each other. These measurements proved that the major source of fluctuations was not the sensing system but the movement of the subject. For all other postures including ‘walking’ , ‘sitting with leg elevated’ , ‘lying straight’ , and ‘lying straight with raised leg’ , the bandage pressure dropped significantly because of reduced physical stress on the pressure sensor. The most significant reported drop was almost 58% of the original value during ‘sitting with leg raised’ as shown in Figure 3(a). The graphs also indicated increased fluctuations in measurements during certain movements wearing a 2-layer bandage as compared to those with a 4-layer bandage. The variations in measured moisture level (Figure 4(a)) over time verified that the measured values were consistent with the dry, wet and intermediate condition of the moisture sensor. Similarly, the body temperature measurement results were also reliable and stable measuring an average value of 35 ± 1°C. Hence, the reliability and consistency of measurements performed on a healthy human volunteer were established during the first trial.

In the second trial, a commonly-used 4-layer bandage system was used to measure sub-bandage pressure near the ankle. The moisture sensor was placed inside a foam dressing through which external fluid was injected in a controlled manner to mimic the wound exudate. This setup was used in this and all following trials. Initial sub-bandage pressure was at 40 mmHg. However, sub-bandage pressure was observed to fluctuate with changing postures as observed during the first trial. This variation was caused by many factors including muscle expansion and contraction associated with various postures, changes in blood flow through the muscles as they expand or contract and the loosening of the bandage layers with movement. It can be observed from Figure 6(b) that with continuous movements, the sub-bandage pressure dropped to almost 50% of the original value. Changes in sub-bandage pressure in such a fashion may adversely affect the healing progress in chronic wounds [43].

In all trials, variations in sub-bandage pressure can be attributed to muscle movement, blood flow direction, and the type and properties of the bandage used. The temperature and moisture measurements did not manifest any dependency on these factors. However, the moisture readings may be strongly affected by the combined effect of the gravity pull and the location of moisture sensor with respect to fluid entry point. In a clinical scenario, a consistent rise in measured temperature could mean infection at wound site. A gradual decrease in moisture level might indicate the start of healing and the opposite might mean the worsening of healing state. A consistently increasing sub-bandage pressure could be a sign of infection or excessive swelling in the limb, and a gradual decrease in sub-bandage pressure might mean loosening of the bandage layers.

Using this device, a clinician would be able to visualize the state of healing of a chronic wound remotely without disturbing the wound, a phenomenon which was never possible in chronic wound management before. However, the device needs further miniaturization and performance improvements, such as enhancing measurement resolutions, in order to have a significant impact on chronic wound monitoring. Although, the moisture and temperature sensors could be used for any wound, however, the pressure sensing capability is useful only for venous leg ulcers. The device works with an external battery which needs to be attached at all times. The estimated cost of the proposed sensing system is around $40 including sensors ($10), circuit components ($20), and flexible circuit manufacturing ($10). This cost could be reduced further if the sensing system is manufactured in bulk quantities and the data is received on a mobile device (i.e. excluding dedicated receiver module). On the other hand, the estimated total cost of the device could potentially exceed the $40-figure owing to regulatory and constraints of the managing health system. The sensing system and sensors are re-usable on a single patient after proper sterilization. The additional cost would be compensated by reduced frequency of dressing changes, reduced nursing time, and reduced use of hospital resources.

## Conclusions

Traditional tools and methods have proven insufficient to effectively monitor the chronic wound healing progress. Inaccuracies in chronic wound measurements are responsible for a significant loss in healthcare budgets e.g. a 30% loss in the US healthcare budget [2]. Engaging an integrated measurement approach (i.e. measuring other critical parameters [16] in addition to wound dimensions) is believed to be more effective to provide stronger evidence of healing than using one kind of measurement only. Though, the wound area and volume calculations over time, and 3D surveillance techniques are useful for this purpose, the proposed sensing system would be a valuable addition to these approaches to mitigate the losses incurred by human errors in chronic wound measurements.

In this paper, we have presented a novel wireless chronic monitoring system which is flexible, biocompatible, and reliable in performance. The sensing system has been fabricated on a flexible circuit material, enabling it to adapt to human limb contours. Low-power profile of the sensing device enables it for being operated continuously under a compression bandage over a longer time period (e.g. 3–4 days) without any disturbance to the bandaged wound. Battery life-time can be further enhanced (e.g. 2–3 weeks) by reducing the frequency of wound data transmission. An effective data transmission range of 4–5 m would enable clinicians and nurses to visualize the current wound state from a distance in a hospital environment.

Experimental results on a healthy human volunteer ascertained that the sensing system was capable of accurately measuring the instantaneous changes in sub-bandage pressure, moisture, and temperature under compression bandages and dressings. Gradual changes in measured wound parameters could reveal the status of healing progress e.g. a consistent rise in sub-bandage pressure might be an indication of infection or swelling. However, the device needs to be tested on patients with chronic wounds to analyze its performance in real environment. The device needs to be tested further even after successful clinical trials to determine its clinical and financial impacts on chronic wound management. The suitability and efficacy of the sensors could be determined during these clinical trials. Nonetheless, the light weight, reliable performance on human limb, flexible and non-invasive structure, low-power consumption and wireless connectivity of the sensing system make it a strong candidate for use in continuous wound sensing and monitoring applications. Future works will incorporate miniaturization of the telemetric sensing system, and its communication with smart phones and display of information on screens using a smart phone application. The system would also incorporate WiFi or 3G communication technologies to share the measured wound parameters to the clinician remotely. This would enable clinicians to gather information on the state of a chronic wound while the patient stays at home.

## Abbreviations

ADC: Analog to digital converter
CRC: Cooperative research center
GHz: Giga Hertz
ID: Identification
LCD: Liquid crystal display
MHz: Mega Hertz
RF: Radio frequency
RFID: Radio frequency identification
RSSI: Received signal strength indicator
RX: Receiver
SD: Standard deviation
SoC: System on chip
TX: Transmitter
